# Sunshine and the cardiovascular benefits – a dose of sunshine!

**Published:** 2010-06

**Authors:** John L Straughan

## God said, ‘Let there be light’, and there was light. (Gen.1:3)

Without sunlight, there would be no life as we know it. Early human societies rightly paid homage to the sun. Many millennia later, and for a host of different reasons, we are again regarding our sun with special interest. That exposure to sunlight is capable of generating a variety of beneficial effects in our skin is an area of exciting discovery – not least for the cardiovascular practitioner.

## Vitamin D from sunshine

Rickets is making a comeback in many parts of the world, even in so-called civilised and fully westernised countries. That sunlight impinging on the skins of humans generates an anti-rachitic vitamin was discovered some 90 years ago, but ignorance of its vital functions and fear of skin neoplasms from sun exposure have brought about the tragic increase in rickets.

In the late 1990s, John Jacob Cannell, an exemplary social activist, began working as a psychiatrist at Atascadero State Hospital, where quite serendipitously he noted that those of his patients who received UV radiation from a ‘sterilising’ light source, enjoyed far better resistance to respiratory infections and better psychiatric health. This reawakened his interest in clinical nutrition. His nutritional studies led to several discoveries about the overlooked need for vitamin D, and inspired him to create the Vitamin D Council (www.vitamindcouncil.org/) in 2003 to promote the vital need for adequate vitamin D.

Structurally, the various forms of vitamin D are secosteroids, and should be regarded as hormones, as they are generated in our skins, from where they circulate around our bodies. The major metabolic product produced in many organs is calcitriol. This highly active secosteroid hormone targets over 2 000 genes, which is about 10% of the human genome.

Ongoing research continues to implicate vitamin D deficiency as a major predisposing factor in the pathology of at least 17 varieties of cancer, as well as heart disease, stroke, hypertension, autoimmune diseases, diabetes mellitus, depression, chronic pain, osteoarthritis, musculoskeletal instability, osteoporosis, muscle weakness, muscle wasting, birth defects, periodontal disease, predisposition to tuberculosis, other microbial diseases and more. A virtual plethora of data supports the importance of adequate bodily levels of calcidiol in reducing overall morbidity and mortality.1-6 A quick perusal of these open full-text reviews assures us that every bodily system benefits from adequate levels of calcidiol, and that with insufficiency, every bodily system suffers.

It is of great importance that we appreciate that foodstuffs, of whatever sort, are poor providers of this vitamin, even in the modern world where several countries vitamin D-fortify milk and other commodities for human consumption.

In the three years since a contribution to this Journal alerting physicians to the cardiovascular risks of a vitamin D deficit, titled ‘Vitamin D – the forgotten vitamin – and the cardiovascular physician’, 7 the data dealing with vitamin D have burgeoned almost explosively. A quick search in Pubmed using just the term ‘vitamin D’, showed that in February 2010, some seven articles per day were published. Google the term ‘vitamin D’, and we get more than 12 million hits! Nevertheless exposure to vitamin D data via various African clinical journals, which presumably are the most widely read journals on this continent, is decidedly minimal or completely absent.

Unpigmented human skin produces approximately 10 000 to 25 000 IU of vitamin D in response 20 to 30 minutes’ summer sun exposure – 50 times more than the US government’s recommendation of 200 IU per day. Short-wave UVB from sunlight acts on the skin precursor 7-dehydrocholesterol to generate, by a fascinating variety of mechanisms, various forms of vitamin D. Chemically these various forms of vitamin D are secosteroids; i.e., steroids in which one of the bonds in the steroid rings is broken. Therefore, what sunlight generates is a series of secosteroid hormones that circulate in our body and act via different vitamin D receptors (VDRs), which are found in almost every cell.

Calcidiol is the important circulating form that is measured to ascertain adequacy or otherwise. Plasma levels of calcidiol should be > 30 ng/ml, while levels greater than 100 ng/ml are very difficult to obtain. A study of highly sunexposed young people in Hawaii concluded that the highest 25 (OH)D concentration produced by natural UV exposure seems to be approximately 60 ng/ml (150 nmol/l).^8^ The magic of our bodily organisation is that there has never been a reported case of vitamin D intoxication due to excessive sun exposure, such as in lifeguards, sun worshippers, etc. The reason is that once the skin makes enough vitamin D, the sun destroys the excess.

In 2003, Gomez produced evidence that secondary hyperparathyroidism is almost non-existent when 25 (OH)D levels exceed 30 ng/ml (requiring 3 000 IU/day).^9^ Dr Vieth cited six studies that concluded, if the aim is to keep parathyroid hormone concentrations low, 25 (OH)D levels should exceed 28 ng/ml (70 nmol/l).^10^

Of special significance to Africa is the fact that persons with highly pigmented skin are particularly at risk of vitamin D deficiency, and require at least five times longer sun exposure to generate the same amount of Vitamin D than would be generated in unpigmented skins.

Data presented at the March 2010 scientific sessions of the American College of Cardiology in Atlanta, Georgia, indicate inter alia, that adequate levels of calcidiol provide cardiovascular benefits of the same order as statin therapy or normalisation of blood pressure in hypertensive persons.

Findings from a study presented at the American Heart Association’s scientific conference on 16 November in Orlando, Florida, found that patients with very low levels of vitamin D (calcidiol) were 77% more likely to die, 45% more likely to develop coronary artery disease, and 78% were more likely to have a stroke than patients with normal levels. Patients with very low levels of calcidiol were also twice as likely to develop heart failure compared with those with normal calcidiol levels. Startlingly, insufficient calcidiol levels were associated with almost zero responsiveness to atorvastatin therapy,^11^ and therefore perhaps to other stains as well.

Therefore it is no exaggeration to claim that an increased risk of morbidity and mortality is associated causally with inadequacy of vitamin D.

## Sunshine on the skin generates other vital agents

Sunlight on our skin generates another vital substance – nitric oxide (NO).^12^ While UVB is responsible for the skin production of the vitamin D entities, UVA radiation releases nitric oxide from our skin. The results from the 1970s MRC Hypertension trial showed that sunlight lowered blood pressure, with lowering effects consistently greater in the summer months than in the wintertime. Human skin is found to be a notable store of nitric oxide moieties, and these are readily mobilised by sun exposure, and delivered around our bodies to provide vasodilation and cardiovascular protection.

The extremely short half-life of NO (measured in nanoseconds) is compensated for by giving rise to the formation of other longer-lived metabolites such as nitrites, nitrates and nitrosothiols that are found in the circulation. Our epidermis is rich in sulphydryls, which readily forms nitrosated products, and these are found at concentrations that are notably higher than those in the plasma. UVA mobilisation of these entities can lead to an increase in plasma nitrite levels of 40%.

There is evidence too that vitamin D can enhance the formation of NO from a multiplicity of sources. But also intriguing is the discovery that sunlight-stimulated formation of melatonin modulates the formation and activity of NO.

Hypertension, ischaemic heart disease, stroke, the metabolic syndrome, and type 2 diabetes are major causes of morbidity and mortality, and while excessive sun exposure carries real risks, there would seem to be no real substitutes for regular small doses of sunshine onto our bodies. True, we can supplement with vitamin D_3_ or D_2_, and even supplement with the amino acids L-arginine and L-citrulline to provide endogenous sources of NO-type moieties, but are we then mimicking all that sunlight can do? Unlikely!

Cautious and regular UV exposure may well allow for major reduction in the annual burden of cardiovascular disease, and would be absolutely cost effective. Such exposure should begin in early childhood, and should never be omitted, especially during pregnancies. The prevention of sunburn is essential if we are to gain the real benefits of sun exposure.

Let us rejoice at the availability of free healthful sunshine, and take it all to heart! Cardiovascular clinicians in their praiseworthy holistic approaches to their patients need to ensure that they add the arrows of sunshine to their therapeutic quivers.
